# Effects of *Verbena* and *Polygonum cuspidatum* on growth performance, immune functions, cecal microbiota, and brain metabolites in Sansui ducks

**DOI:** 10.3389/fvets.2025.1615674

**Published:** 2025-07-09

**Authors:** Yongcai Zhu, Qiaoqun Wu, Linli Luo, Shenglin Yang

**Affiliations:** ^1^Key Laboratory of Animal Genetics, Breeding and Reproduction in the Plateau Mountainous Region, Ministry of Education, Guizhou University, Guiyang, China; ^2^School of Animal Technology and Innovation, Institute of Agricultural Technology, Suranaree University of Technology, Nakhon Ratchasima, Thailand

**Keywords:** Verbena, *Polygonum cuspidatum*, duck, immunity, growth performance, brain metabolite, microbiota

## Abstract

*Verbena* and *Polygonum cuspidatum*, recognized for their antioxidant and immunomodulatory properties, have demonstrated potential benefits. However, the specific mechanisms by which these herbs impact poultry health, particularly regarding alterations in gut microbiota and brain metabolite profiles, remain insufficiently investigated. This study aimed to investigate the effects of *Verbena* and *Polygonum cuspidatum* supplementation on the growth performance, immunity, cecal microbiota, and brain metabolites in Sansui ducks. A total of 216 one-day-old ducks were randomly assigned to three treatments for a 35-day trial, each with 6 replicates of 12 ducks. The dietary treatments included a basal diet (T3), a basal diet supplemented with 40 mg/kg *Verbena* (T1), and a basal diet supplemented with 40 mg/kg *Polygonum cuspidatum* (T2). The results showed that both *Verbena* and *Polygonum* treatments significantly improved final body weight (by 3.0 and 4.1%, respectively) and increased serum IgG and IgM levels by 7.4 and 9.0%, and decreased feed conversion rates by 5.4 and 5.0%, respectively, compared to the control (*p* < 0.05). Notably, *Verbena* supplementation significantly increased the relative abundance of *Bacteroidetes* and *Saccharibacteria*, and significantly decreased the relative abundance of *Actinobacteria* compared to the control group (*p* < 0.05). *Polygonum cuspidatum* treatment increased the relative abundance of *Megamonas* compared to the control group (*p* < 0.05). Brain metabolite analysis showed that *Verbena* increased glutamine (Gln) levels by 18.4% and decreased *γ*-aminobutyric acid (GABA), tyrosine (Tyr), and acetylcholine (Ach) by 19.7, 14.6, and 22.5%, respectively (*p* < 0.05). *Polygonum cuspidatum* increased 5-Hydroxyindoleacetic acid (5-HIAA) concentration by 31.2% (*p* < 0.05). Correlation analysis indicated associations between gut microbiota (*Villanella*, *Anaerosporobacter*, *Anaerofustis*, and *Flavonifractor*) changes and brain metabolites (GABA, Ach, and Glutamic [Glu]), suggesting the potential influence of these herbs through the microbiota-gut-brain axis. Supplementation with *Verbena officinalis* and *Polygonum cuspidatum* enhanced growth performance, immunity, and brain neurochemical profiles, potentially through gut microbiota modulation. These herbs show promise as functional feed additives in duck production.

## 1 Introduction

Antibiotics are commonly employed in the poultry industry to enhance growth and improve feed efficiency by promoting intestinal health (1). However, the widespread and prolonged use of antibiotics has led to the rise of resistance, and the presence of antibiotic residues in poultry meat and waste poses direct or indirect risks to both human and animal health, and countries are taking antibiotics more seriously (2). Therefore, these concerns have prompted an increasing interest in finding natural and efficient alternatives to antibiotics, particularly those that can enhance intestinal health and promote growth performance (3).

Natural feed additives, including herbal supplements, have shown promise as potential alternatives to antibiotics in poultry. Previous studies have indicated that the beneficial effects of Chinese herbs on poultry include promoting growth (4), enhancing immunity (5), and regulating intestinal microbiota (6). Among these, *Verbena* and *Polygonum cuspidatum*, two Chinese herbs, are also utilized as feed supplements owing to their distinct biological activities, including antioxidant, anti-inflammatory, antibacterial, and growth-promoting properties. Studies have indicated that bioactive compounds in these herbs, including flavonoids, verbascoside (from *Verbena*), and resveratrol (from *Polygonum cuspidatum*), play crucial roles in promoting growth, improving feed efficiency, and maintaining intestinal health in animals (7). The flavonoids in hawthorn have pharmacological effects, including improving intestinal flora and preventing neuronal cytopathy, hypertension, hyperlipidemia, hyperglycemia, and tumors (8). The main active components of *Verbena* are polyphenols, in particular verbascoside, that improve gut health in poultry, with a stabilizing effect on gut microbiota and a positive effect on productive parameters (9). Its neuroprotective effects suggest a potential influence on neurotransmitter pathways relevant to the gut-brain axis. Previous studies have shown that pigs fed a diet enriched with verbascoside had improved growth and feed efficiency (10). Additionally, studies have shown that dietary resveratrol supplementation can enhance the immune response, modulate the intestinal microbiota, and improve feed efficiency in poultry (11). Moreover, resveratrol has been reported to influence gut microbiota composition and increase central serotonin and dopamine levels, further supporting its relevance in gut-brain axis modulation (12). However, the precise mechanisms through which these herbal supplements exert their effects on poultry performance and gut health, particularly the connection between gut microbiota and brain metabolites, remain underexplored.

The bidirectional communication between the gut microbiota and the brain is termed the microbiota–gut–brain axis. The gut microbiota is known to influence brain function through the production of metabolites, which in turn affect various physiological processes, including immune modulation, behavior, and metabolism (13). While studies have primarily focused on the gut’s role in influencing metabolic processes, less is known about how dietary supplements like *Verbena* and *Polygonum cuspidatum* affect this microbiome-brain communication. It is hypothesized that herbal supplementation could impact the composition of the gut microbiota, which may subsequently alter the levels of brain metabolites involved in neurotransmission and neurophysiological processes. Key neurotransmitters implicated in this axis include GABA, dopamine (DA), norepinephrine (NE), serotonin (5-HT), and histamine (His), which are either produced or modulated by intestinal microbes and have been shown to affect stress responses and cognitive functions (14). Despite growing interest in this field, the precise mechanisms linking microbial shifts to neurochemical changes remain poorly defined, particularly in poultry models.

Indigenous poultry breeds such as the Sansui duck offer a promising model for gut-brain axis studies due to their distinctive gastrointestinal physiology and heightened sensitivity to dietary modulation. Moreover, their stable genetic background and consistent performance under varied feeding conditions make them suitable for investigating the interplay between herbal supplements, gut microbiota, and brain metabolites. By combining growth performance data, cecal microbiota analysis, and brain metabolite profiling, this research seeks to uncover the complex interplay between diet, gut ecology, and brain biochemistry, ultimately contributing novel insights into the potential of herbal supplementation as a natural alternative to antibiotics in poultry production. We hypothesize that dietary supplementation with *Verbena officinalis* and *Polygonum cuspidatum* can improve growth performance and immune function in Sansui ducks by modulating cecal microbiota composition and altering brain neurotransmitter-related metabolites, thereby influencing the microbiota–gut–brain axis.

## 2 Materials and methods

### 2.1 Animal ethics

The experiment was carried out at the Guizhou University farm in accordance with the approved protocol, as authorized by the Animal Ethics Committee of Guizhou University (Guiyang, China; No. EAE-GZU-2022-E032).

### 2.2 Birds, experimental design, and diets

Herbs (*Verbena* and *Polygonum cuspidatum* powder) were purchased from Ju Chun Tang Chinese Herbal Medicine Sales Co., LTD., Bozhou City, Anhui Province, China. The feeding process was mixed in powder form. *Verbena* (powder obtained from dried *Verbena* roots) is rich in flavonoids and verbascoside (15). *Polygonum cuspidatum* (powder obtained from dried *Polygonum cuspidatum* roots) is rich in resveratrol (16). Both herbs were ground to pass through a 100-mesh sieve before being uniformly mixed into the diet. No further chemical standardization or quantification of the active compounds was performed in this study. A total of 216 female Sansui ducks (1-day-old) with similar body weight were supplied by Sanyuan Agricultural Development Co., Ltd., Guizhou, China. Sansui ducks are an indigenous Chinese breed known for stable genetics and sensitivity to dietary interventions, and randomly divided into three treatments of 12 ducks each (6 replicates/treatment), housed in individual cages (100 cm length × 100 cm width × 120 cm height). The dietary treatments were the control (T3) group: a basal diet; *Verbena* (T1) group: a basal diet with 40 mg/kg *Verbena*; *Polygonum cuspidatum* (T2) group: a basal diet with 40 mg/kg *Polygonum cuspidatum*. Ambient temperature was maintained at 33 ± 2°C during the first week and gradually reduced to 25°C by the end of the experiment. Humidity ranged between 55 and 70%. The composition and nutrient levels of the diets are shown in Table 1. The 40 mg/kg dose was based on an unpublished graduate thesis by the first author, which identified this level as optimal for improving growth, immunity, and gut microbiota in Sansui ducks. Diets were formulated to meet nutritional requirements according to the recommendations of the National Research Council (17), containing 2,800 kcal of metabolizable energy/kg and 15% crude protein, and were maintained on a 16-h light cycle daily, with water available ad libitum. At the end of 35 days of age, six ducks were randomly selected from each of the three groups following a 12-h fasting period. The pen number and individual body weight of each duck were recorded prior to their slaughter via cervical dislocation. Approximately 3 mL of blood was collected from the subwing vein using a vacuum coagulation tube, allowed to stand at 25°C for 2 h, and then placed on ice. After coagulation, the serum was separated and centrifuged at 3,000 rpm for 10 min to eliminate impurities. The resulting supernatant was transferred to a clean centrifuge tube and stored at −20°C for further analysis. Cecal content and brain tissue samples were collected from the same ducks immediately after slaughter on day 35, ensuring that microbial and neurochemical data used for correlation analysis originated from the same individuals. Whole brains were isolated, rinsed in ice-cold phosphate-buffered saline (PBS) to remove blood and other contaminants, immediately frozen in liquid nitrogen, and then stored at −80°C for metabolite analysis. For metabolite analysis. The entire brain tissue was homogenized, and no dissection of specific brain regions (e.g., hypothalamus) was performed. The cecum contents samples were collected, quickly frozen in liquid nitrogen, and stored in a refrigerator at −20°C for subsequent analyses.

**Table 1:** Composition and nutrient levels of the experimental diets (air-dry basis).

Ingredients		Nutrient level^b^	
Corn	55.75	CP (%)	18.10
Soybean meal	27.40	ME (MJ/kg)	10.65
Wheat bran	1.50	Crude fiber (%)	3.07
RaPeseed cake	4.00	Ca (%)	3.37
CaHPO_4_	2.75	P (%)	0.63
Limestone	7.25	Lysine (%)	0.92
NaCl	0.35	Methionine (%)	0.27
Premix^a^	1.00		
Total	100.00		

a Pemix provided per kilogram of diet: vitamin A, 4000 IU, vitamin E, 20 mg; vitamin K3, 2 mg; vitamin B1, 3.5 mg; vitamin B12, 0.01 mg; niacin, 50 mg; folic acid, 1.0 mg; Cu, 10 mg; Fe, 80 mg; Mn, 60 mg; Zn, 60 mg; I, 0.4 mg; Se, 0.2 mg; Calcium Pantotherate, 10 mg; Pyridoxol, 2.5 mg; biotin, 0.1 mg.

b ME is calculated, and the rest are measured values.

### 2.3 Determination of immunoglobulin levels in the serum

An enzyme-linked immunosorbent assay (ELISA) was used to determine the levels of immunoglobulin (Ig)A, IgG, and IgM, levels in the serum. All commercial ELISA kits used for testing were purchased from Nanjing Jiancheng Bioengineering Institute. The catalog numbers were: IgA – H197, IgG – H164, and IgM – H195. Assays were performed according to the manufacturer’s instructions. Briefly, 50 μL of serum samples or standards were added to microplate wells pre-coated with specific antibodies and incubated at 37°C for 1 h. After washing, horseradish peroxidase (HRP)-conjugated antibodies were added and incubated for an additional 30 min. The wells were washed again, followed by the addition of TMB substrate solution. After a 15-min incubation in the dark, the stop solution was added, and absorbance was read at 450 nm using a microplate reader (BioTek Instruments, USA). Immunoglobulin concentrations were calculated based on standard curves prepared for each analyte.

### 2.4 Bacterial DNA extraction and 16S rRNA gene sequencing

DNA was extracted from the contents of the cecum using the QIAamp DNA Stool Mini Kit (catalog NO. 51504, Qiagen, CA, USA) following the manufacturer’s instructions. The V3-V4 region of the bacterial 16S rRNA gene was PCR amplified using primers 338F (5′-ACTCCTACGGGAGGCAGCA-3′) and 806R (5′-GGACTACHVGGGTWTCTAAT-3′). All PCR products have been recovered using the AxyPrep DNA gel recovery kit and quantified using the FTC-3000TM real-time PCR instrument. After obtaining the DNA fragment sequence, the QIAamp DNA Stool Mini Kit was used to construct the library, Illumina’s Miseq PE300 platform was used for sequencing, and the sequencing was completed at Shanghai Weiji Biotechnology Co., Ltd. (Shanghai, China). For 16S rRNA gene analysis, raw sequence data were subjected to quality control using QIIME2^1^, which removed low-quality sequences and potential contaminants. UPARSE software was used to cluster operational taxonomic units (OTUs) based on 97% sequence similarity. Alpha diversity of the microbial communities was assessed using Chao1, Shannon, and Simpson indices in QIIME2. Beta diversity was evaluated through principal coordinate analysis (PCoA). Differences in relative bacterial abundance were determined using the nonparametric Kruskal-Wallis sum-rank test. Bacterial biomarkers distinguishing the microbial communities across all groups were identified using linear discriminant analysis (LDA) effect size (LEfSe) (LDA > 2.5, *p* < 0.05). The co-occurrence of microbial communities was analyzed for the top 40 genera based on significant Spearman correlations (*p* < 0.05).

### 2.5 Brain metabolite analysis

Accurately weigh 15 mg of sample into a 2 mL EP tube, accurately add 200 μL of 10% formic acid methanol solution-ddH_2_O (1:1. V/V) solution, add 50 mg of glass beads; put it into a high-throughput tissue grinder and oscillate at 60 Hz for 1 min, repeat twice; centrifuge at 12000 rpm and 4°C for 5 min, take 50 μL of supernatant, accurately add 50 μL of dual isotope internal standard with a concentration of 100 ppb, vortex and oscillate for 30 s, filter the supernatant through a 0.22 μm membrane, and add the filtrate to the detection bottle (detection of low-content substances). Take 10 μL of the original supernatant, add 490 μL of 10% formic acid methanol solution-ddH_2_O (1:1. V/V) solution, vortex and oscillate for 30 s, take 100 μL of the diluted sample, add 100 μL of 100 ppb dual isotope internal standard, vortex and oscillate for 30 s, filter the supernatant through a 0.22 μm membrane, and add the filtrate to the detection bottle (detection of high-content substances) vial for liquid chromatography-mass spectrometry (LC–MS) analysis. Weigh appropriate amounts of 10 brain metabolites (GABA, Gln, Glu, His, L-Histidine [L-His], Tyr, Tryptamine [Trp], Ach, NE, and 5-HIAA) standards and prepare single standard stock solutions with 10% formic acid in methanol. Take appropriate amounts of each stock solution to prepare mixed standards, dilute each to the appropriate concentration with 10% formic acid in methanol, and prepare working standard solutions. For LC–MS analysis, chromatographic conditions: chromatographic column: ACQUITY UPLC BEH C18 column (2.1 × 100 mm, 1.7 μm, Waters, USA), injection volume 5 μL, column temperature 40°C, mobile phase A-10% methanol–water (containing 0.1% formic acid), B-50% methanol–water (containing 0.1% formic acid). Gradient elution conditions are 0–1 min, 20–100% B; 1–7 min, 100% B; 7–7.5 min, 100–20% B; 7.5–11 min, 20% B. Flow rate 0.4 mL/min. Mass spectrometry conditions: electrospray ionization (ESI) source, positive ionization mode. The ion source temperature is 500°C, the ion source voltage is 5,500 V, the collision gas is 6 psi, the curtain gas is 30 psi, and the nebulizer gas and auxiliary gas are 50 psi. Multiple reaction monitoring (MRM) scanning was used.

### 2.6 Statistical analysis

Data for growth performance, immunoglobulin levels, and brain metabolite concentrations were analysed using SPSS software (version 27.0). All data values were stated as mean ± SEM. One-way analysis of variance (ANOVA) and Tukey’s *post hoc* test were employed to determine the significance of mean differences. *p*-values were considered statistically different at *p* < 0.05. Alpha diversity analysis was calculated based on the Chao index, Shannon index, and Simpson index using QIIME2 (v1.9.1). Beta diversity was calculated based on the unweighted UniFrac distance, and statistical comparisons among groups were performed with permutational ANOVA. Figures were generated in GraphPad Prism 10.0 software (GraphPad Software Inc., San Diego, CA). Spearman rank correlation analysis assessed the relationship between microbiota and other brain metabolites.

## 3 Results

### 3.1 Growth performance and serum immunoglobulins

The effect of *Verbena* and *Polygonum cuspidatum* supplementation in duck diets on growth performance and serum immunoglobulins is shown in Table 2. Dietary supplementation with *Verbena* and *Polygonum cuspidatum* significantly increased final body weight, as well as IgG and IgM levels, while decreasing the feed conversion ratio compared to the ducks fed with the control diets (*p* < 0.05).

**Table 2:** Effect of *Verbena* and *Polygonum cuspidatum* on growth performance and plasma immunoglobulins of Sansui ducks.

Item	Groups			*P-*value
	T 3(Control)	T1 (*Verbena*)	T2 (*Polygonum cuspidatum*)	
Body weight, g/duck	205.67^b^	211.90^a^	214.11^a^	<0.001
1 to 35 d				
Feed intake, g/ducks	790.40	798.61	792.32	0.307
1 to 35 d				
Feed conversion ratio	2.52^a^	2.39^b^	2.40^b^	0.001
1 to 35 d				
Serum immunoglobulins				
IgA (g/L)	0.82	0.85	0.92	0.112
IgG (g/L)	1.22^b^	1.31^a^	1.33^a^	0.004
IgM (g/L)	0.93^b^	1.07^a^	1.13^a^	0.038

T3: feed with the basic diet; T2: feed with basic diet and 40 mg/kg *Polygonum cuspidatum*; T1: feed with basic diet and 40 mg/kg *Verbena*. ^a, b^ Means within each row with different superscripts are significantly different (*P* < 0.05).

### 3.2 Microbial composition of the cecum

**Figure 1:**
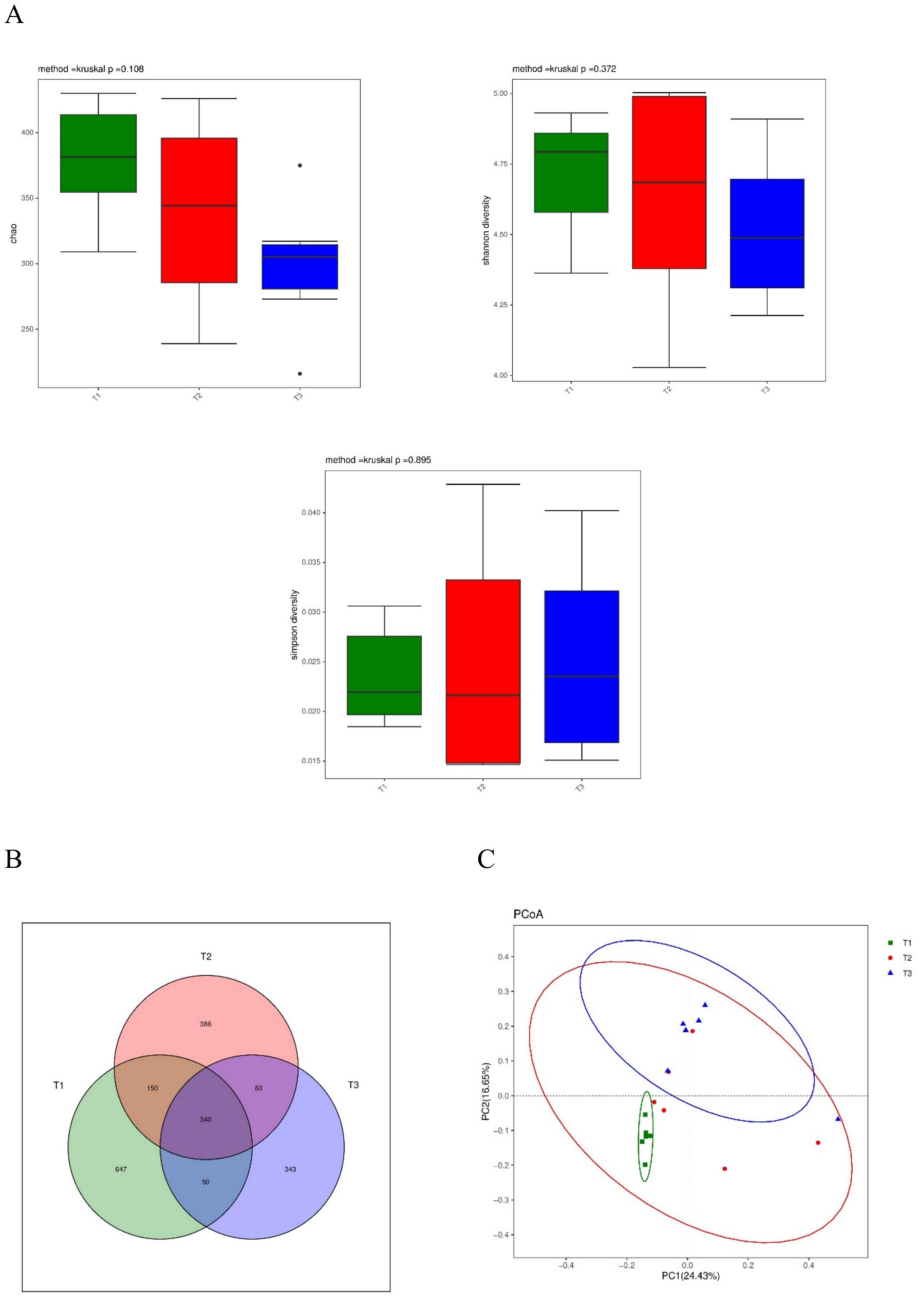
Effect of dietary supplementation with *Verbena* and *Polygonum cuspidatum* on diversity of the cecal microbiota in Sansui ducks. **(A)** Chao index, Shannon index, and Simpson index. **(B)** A Venn diagram based on the OTU level. **(C)** Principal coordinate analysis (PCoA) based on Unweighted UniFrac. T3: feed with the basic diet; T2: feed with basic diet and 40 mg/kg *Polygonum cuspidatum*; T1: feed with basic diet and 40 mg/kg *Verbena*.

The composition of the microbial community in the cecal digesta using 16S rRNA amplicon sequencing is shown in Figure 1. The Chao index estimates the species’ richness (i.e., the number of species present) in a treatment. Simpson’s index measures microbial richness or evenness, while Shannon entropy measures species richness and the community’s evenness in a sample or within a treatment. The Chao index, Simpson index, and Shannon index in the alpha diversity among the groups were not significantly different (Figure 1A). The Venn diagram (Figure 1B) shows that 3 groups contained a total of 340 shared OTUs, while the T3, T2, and T1 groups had 343, 386, and 647 unique OTUs, respectively. The PCoA by Unweighted UniFrac distance indicated that the control group was separated from the other groups (Figure 1C).

**Figure 2:**
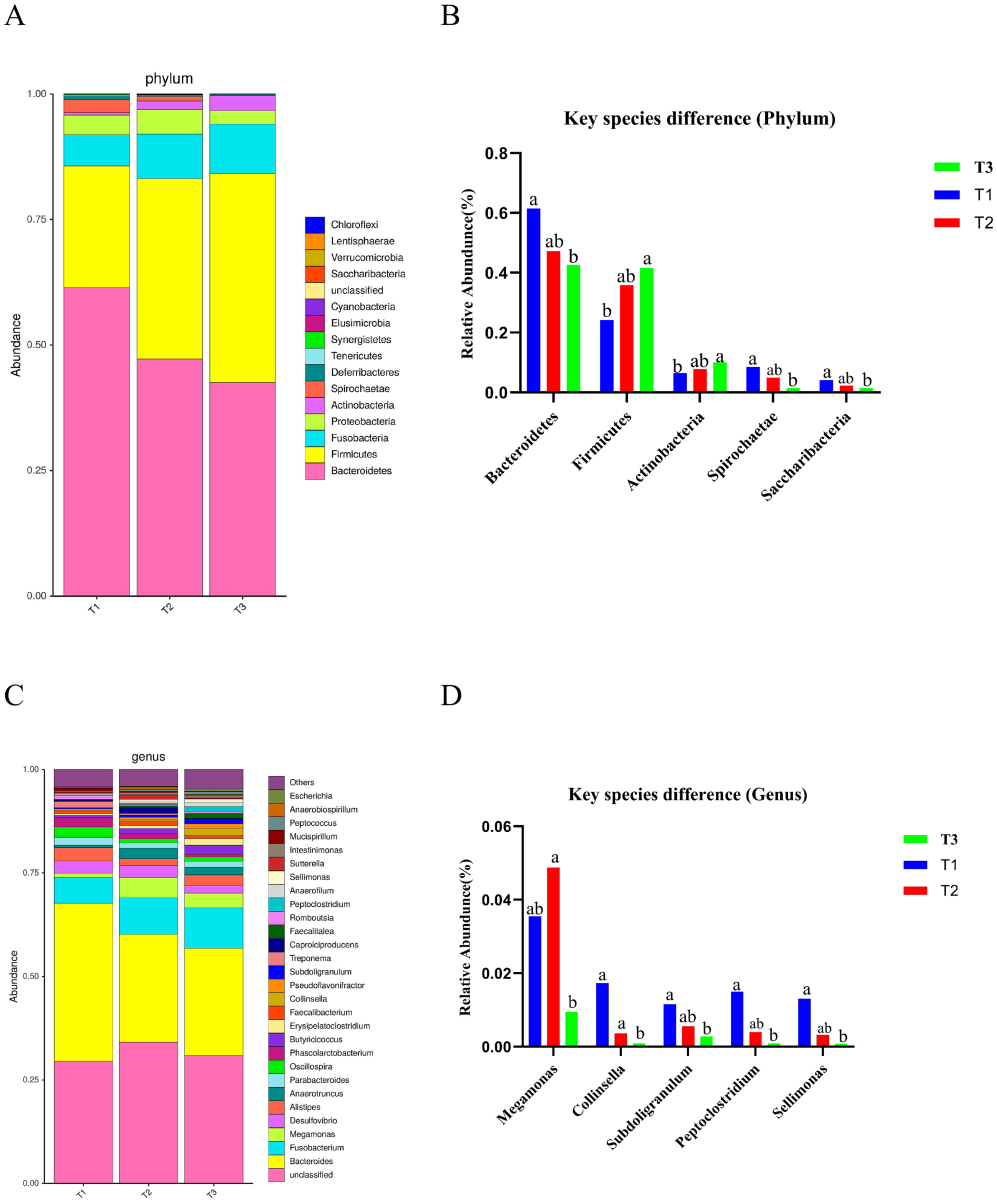
Effect of dietary supplementation with *Verbena* and *Polygonum cuspidatum* on composition of the cecal microbiota in Sansui ducks. **(A)** Microbial composition at the phylum level. **(B)** Significantly abundant microbiota at the phylum level. **(C)** Microbial composition at the genus level. **(D)** Significantly abundant microbiota at the genus level. ^a,b^ indicates significant differences (*p* ≤ 0.05). T3: feed with the basic diet; T2: feed with basic diet and 40 mg/kg *Polygonum cuspidatum*; T1: feed with basic diet and 40 mg/kg *Verbena*.

Additionally, *Bacteroidetes* and *Firmicutes* were the major phylum species across all groups (Figure 2A). Further analysis of the phylum composition of the top 25 ranked groups showed that a total of 5 differential bacteria were identified in each group, with a significant increase in the relative abundance of *Bacteroidetes*, *Spirochaetae* and *Saccharibacteria* in the *Verbena* supplemented group compared to control group, and a significant decrease in the relative abundance of *Fimicutes* and *Actinobacteria* (*p* < 0.05) (Figure 2B). Taxon-based analysis at the genus level revealed *Bacteroides*, *Fusobacterium*, and *Megamonas* as the predominant genera (Figure 2C). The *Collinsella*, *Subdoligranulum*, *Peptoclostridium*, and *Sellimonas* were significantly enriched in the *Verbena* supplemented group at the genus level as compared to the control group (*p* < 0.05), while supplementation with *Polygonum cuspidatum* significantly increased the relative abundance of *Megamonas* compared to the control group (*p* < 0.05) (Figure 2D).

### 3.3 LEfSe analysis of species

**Figure 3:**
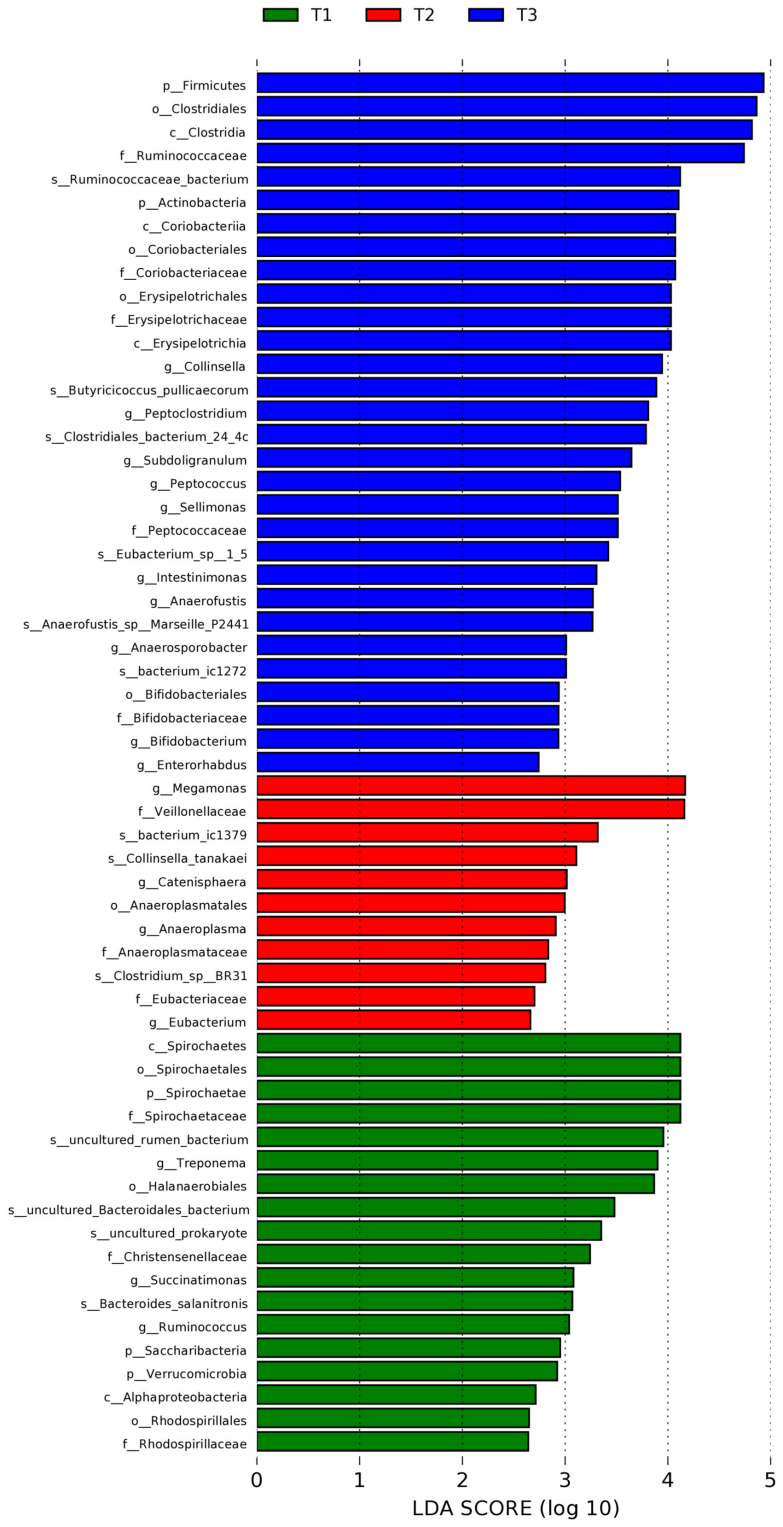
Linear discriminant analysis (LDA) effect size (LEfSe) of intestinal microbiota (LDA > 2.5, *p* < 0.05). T3: feed with the basic diet; T2: feed with basic diet and 40 mg/kg *Polygonum cuspidatum*; T1: feed with basic diet and 40 mg/kg *Verbena*.

To identify the specific bacteria that were characteristic of the 3 groups, LEfSe was used (expressed as values of linear discriminant analysis) in further evaluating the differences in bacterial composition among the different dietary treatments (Figure 3). At the phylum level, a great abundance of *Firmicutes* and *Actinobacteria* in the control (T3) group, and *Spirochaetae*, *Saccharibacteria*, and *Verrucomicrobia* in the *Verbena* supplemented (T1) group were detected. At the genus level, *Collinsella*, *Peptoclostridium*, *Subdoligranulum*, *Sellimonas*, *Intestinmonas*, *Anaerofustis*, *Anaerosporobacter*, *Bifidobacteriales*, and *Enterorhabdus* in the control (T3) group, and *Megamonas*, *Catenisphaera*, *Anaeroplasma*, and *Eubacterium* in the *Polygonum cuspidatum* (T2) group, and *Treponema*, *Succinatimonas*, and *Ruminoccus* in the *Verbena* supplemented (T1) group were detected.

### 3.4 Effects of *Verbena* and *Polygonum cuspidatum* on the level of the brain metabolites of Sansui ducks

**Figure 4:**
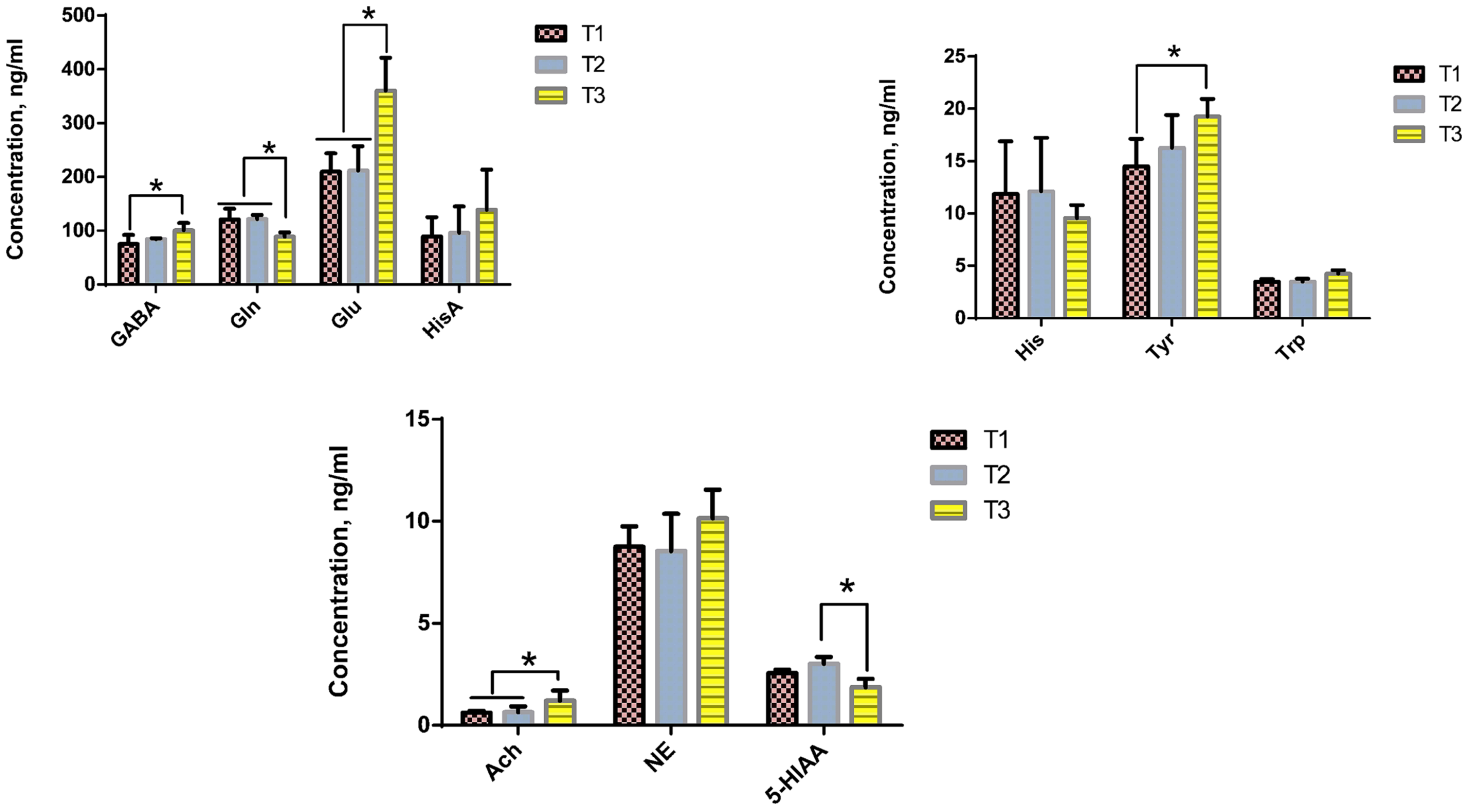
Effect of dietary supplementation with *Verbena* and *Polygonum cuspidatum* on brain metabolites in Sansui ducks. *Indicates significant differences (*p* ≤ 0.05). T3: feed with the basic diet; T2: feed with basic diet and 40 mg/kg *Polygonum cuspidatum*; T1: feed with basic diet and 40 mg/kg *Verbena*.

The effect of dietary *Verbena* and *Polygonum cuspidatum* supplementation on the concentrations of brain metabolites in Sansui ducks is presented in Figure 4. Tukey’s multiple comparison tests indicated that dietary supplementation with *Verbena* significantly reduced the GABA and Tyr concentrations (*p* < 0.05), and both *Verbena* and *Polygonum cuspidatum* supplementation significantly increased the Gln concentrations compared to the control group (*p* < 0.05). Based on Tukey’s multiple comparison tests, dietary supplementation with *Verbena* and *Polygonum cuspidatum* significantly decreased Glu and Ach concentrations (*p* < 0.05), while *Polygonum cuspidatum* supplementation significantly increased 5-HIAA concentration compared to the control group (*p* < 0.05).

### 3.5 Correlation between brain metabolite concentrations and cecal microbiota in Sansui ducks

**Figure 5:**
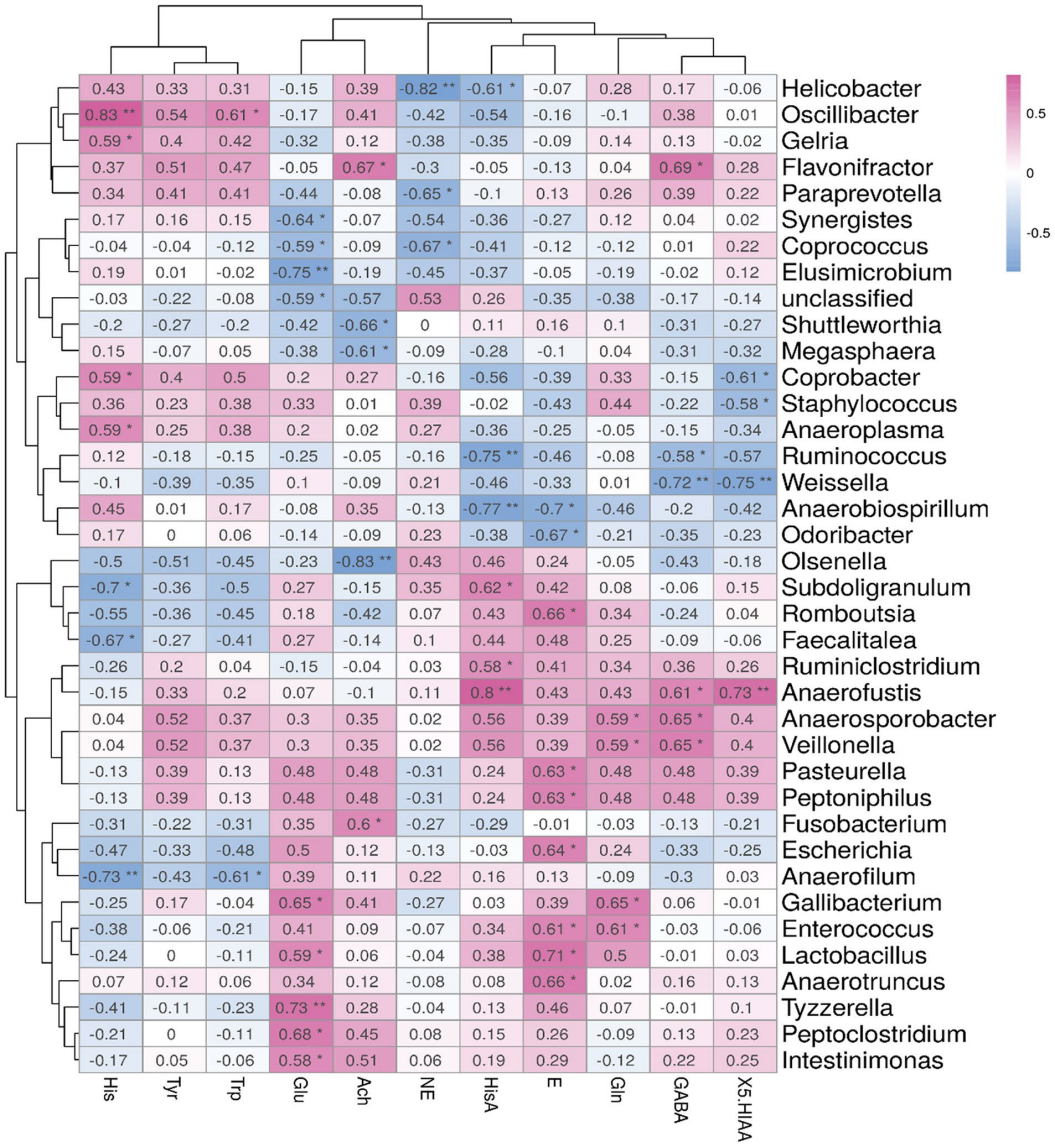
Spearman’s correlation analysis between the abundances of cecal microbiota and brain metabolite concentrations. Significant correlations are noted by 0.01 < *p* ≤ 0.05*, 0.001 < *p* ≤ 0.01**, Red represents positive correlation and blue represents negative correlation.

To find out the correlation between brain metabolites and cecal microbiota, we performed a Spearman correlation analysis between the top 40 differential bacteria and brain metabolite concentrations in Sansui ducks treated with *Verbena* and *Polygonum cuspidatum,* as shown in Figure 5. The level of Glu showed a positive correlation with 5 bacterial genera (*Intestinimonas, Peptoclostridium, Tyzzerella, Lactobacillus*, and *Gallibacterium*) and a negative correlation with 3 major bacterial genera (*Elusimicrobium, Coprococcus*, and *Synergistes*). The level of Ach showed a positive correlation with 2 main bacterial genera (*Fusobacterium* and *Flavonifractor*) and a negative correlation with 3 bacterial genera (*Olsenella, Megasphaera,* and *Shuttleworthia*). The level of Gln had no negative correlation with all bacterial genera but showed a positive correlation with 4 bacterial genera (*Enterococcus, Gallibacterium, Veillonella,* and *Anaerosporobacter*). Moreover, the GABA level showed a positive correlation with 4 bacterial genera (*Veillonella, Anaerosporobacter, Anaerofustis,* and *Flavonifractor*) and a negative correlation with *Weissella* and *Ruminococcus*. The level of 5-HIAA showed a positive correlation with *Anaerofustis* and a negative correlation with 3 bacterial genera (*Weissella, Staphylococcus,* and *Coprobacter*).

## 4 Discussion

In traditional herbal medicine, *Verbena* and *Polygonum cuspidatum* are polyphenolic plants that have been reported to possess antioxidant and immunostimulant properties (18). Our results showed that ducks fed *Verbena* and *Polygonum cuspidatum* significantly increased final body weight and decreased feed conversion ratio, indicating improved feed efficiency. These improvements in growth performance align with previous studies on *Verbena* and *Polygonum cuspidatum* bioactive substances, such as flavonoids and verbascoside from *Verbena*, and resveratrol from *Polygonum cuspidatum*, which are known to enhance nutrient absorption, modulate gut health, and reduce oxidative stress in poultry (7), which also shows that it might be ascribed to these bioactive components from *Verbena* and *Polygonum cuspidatum* improved overall growth performance in poultry. Some studies reported that supplementation of 400 mg/kg resveratrol in diets led to an improvement in the growth performance of broiler chickens (19). Previous research has supplemented the diets of 25 to 42-day-old broiler chickens with *Verbena* leaf powder at 0.5 and 1.0%. At the higher dose, verbena leaf powder increased average weight gain and feed intake, while decreasing feed conversion ratio, compared to the control group (18). However, although *Polygonum cuspidatum* and *Verbena* are known to contain bioactive constituents such as resveratrol and verbascoside, we did not quantify these compounds in this study.

Owing to the crucial role in the immune system, immunoglobulins are commonly used to evaluate the immune status of animals, and three main types, IgA, IgG, and IgM, are present in birds, which could resist the intrusion of a variety of pathogens and toxins (20). The IgG is the major avian systemic antibody that acts upon infection with an immunoglobulin that occurs as a second antibody reaction after IgM production (21). Our results showed that *Verbena* and *Polygonum cuspidatum* enhanced the immune status, evidenced by elevated serum levels of IgG and IgM, suggesting that these herbs may exert immunomodulatory effects. Similar increases in IgG and IgM concentrations upon treatment with *Lemon Verbena* and resveratrol have been confirmed in broilers (20). In addition, a study showed that dietary supplementation of 300 or 600 mg resveratrol/kg enhanced total Ig and IgG in the broilers, compared to the control treatment at d 22 (22). The bioactive components from *Verbena* and *Polygonum cuspidatum* have been shown to promote humoral immunity by activating lymphocyte proliferation and antibody production (23). Although IgA levels did not change significantly, the increase in IgG and IgM supports the hypothesis that these herbal supplements may enhance mucosal and systemic immune responses, possibly contributing to better disease resistance and growth.

It is very important to study fecal microbiota for the growth and health of animals (24). The Chao1, Shannon, and Simpson indices reflect the richness and diversity of microbiota. In our study, despite no significant differences in alpha diversity indices (Chao1, Shannon, and Simpson), the beta diversity (PCoA analysis) clearly showed that the microbial communities in the control group were distinctly separated from those in the *Verbena* and *Polygonum cuspidatum* groups. These observations are consistent with those of several studies on resveratrol supplementation (22). These findings suggest that *Verbena* and *Polygonum cuspidatum* supplementation altered the composition of the cecal microbiota without substantially affecting overall diversity. The lack of significant changes in alpha diversity may be attributed to several factors. First, it is possible that the 35-day duration of the experiment, while appropriate for capturing early-life microbial shifts, may not be long enough to induce measurable changes in species richness or evenness, particularly if the existing microbiota is resilient or functionally redundant. Previous studies have shown that microbial colonization and community restructuring occur rapidly during the early life of poultry and can be influenced by various factors, including diet and environmental exposure (25, 26). Second, the gut microbiota of ducks during the early growth phase may possess a relatively stable core microbial structure that resists substantial disruption from short-term dietary interventions. This microbial stability could serve a protective function, allowing for compositional shifts (as seen in beta diversity) without compromising overall diversity. Therefore, future studies extending beyond this early-life window are needed to determine whether the observed compositional shifts lead to sustained changes in microbial ecology or neurobiological function.

Microbiota in the gut play an important role in digestion, metabolism, immunity, and pathogen defense in animals (27). In this study, *Firmicutes* and *Bacteroidetes* were identified as the dominant phyla in the cecal contents of Sansui ducks, a result that aligns with previous reports on the microbiota of ducks (28). Studies have demonstrated that *Firmicutes* negatively affect growth performance and intestinal barrier function, but *Bacteroidetes* exert a positive influence in these areas (29). *Bacteroidetes* are crucial for carbohydrate fermentation and demonstrate inhibitory effects on pathogen colonization (30). Furthermore, the *Bacteroidetes*/*Firmicutes* ratio serves as a key indicator of microbiota functionality (31). Our results indicated that supplementation with *Verbena* significantly increased *Bacteroidetes* abundance, while decreasing *Firmicutes* abundance compared to the control group, thereby increasing the *Bacteroidetes*/*Firmicutes* ratio, a result that is consistent with previous studies (32). Given the increased *Bacteroidetes*/*Firmicutes* ratio, alongside the elevated final body weight and decreased feed conversion ratio, we proposed that the changes in the *Bacteroidetes*/*Firmicutes* ratio induced by *Verbena* supplementation may be responsible for the observed improvement in growth performance. Moreover, the study showed that rumen-protected lysine changed the relative abundance of *Firmicutes* and *Proteobacteria*, indicating that rumen-protected lysine affects the microbial community in the hindgut of dairy cows (33). These findings suggest that *Verbena* and *Polygonum cuspidatum* affect the microbial community in the cecum of ducks. In addition, supplementation with *Verbena* significantly increased *Saccharibacteria* abundance, while decreasing *Actinobacteria* abundance compared to the control group. *Actinobacteria* are associated with dysbiosis and inflammation (34). On the other hand, *Actinobacteria* have the ability to effectively degrade cellulose, hemicellulose, and lignin (35), indicating supplementation with *Verbena* enhances immunity and feed efficiency. To our best knowledge, the role of *Saccharibacteria* (a candidate bacterial phylum) in the duck intestinal microbiota remains inadequately understood (36). At the genus level, supplementation with *Verbena* significantly increased the abundance of *Collinsella*, *Subdoligranulum*, *Peptoclostridium*, and *Sellimonas*, while supplementation with *Polygonum cuspidatum* significantly increased *Megamonas* abundance, compared to the control group in this study, which is consistent with previous findings (37). *Collinsella* primarily produces gases in the intestine, a process that has been linked to abnormal lipid metabolism and type 2 diabetes (38). *Subdoligranulum* belongs to the Ruminococcaceae family. Previous studies have shown that *Subdoligranulum* has the ability to produce butyrate, which plays a key role in promoting intestinal health by supplying energy to host cells and maintaining the integrity of the intestinal barrier (38). Similarly, studies suggested that *Peptoclostridium* plays a role in modulating immune responses, reducing inflammation, and stimulating the production of butyrate, a key factor in maintaining intestinal health (39). *Megamonas* has also been shown to utilize amino acids or carbohydrates to produce acetic acid, which plays a crucial role in intestinal energy supply, maintenance of the intestinal mucosal barrier, and regulation of intestinal motility (40). These findings indicated that supplementation with *Verbena* or *Polygonum cuspidatum* promotes intestinal microbiota balance by increasing the abundance of beneficial bacteria and decreasing pathogenic bacterial populations, thereby enhancing intestinal health and indirectly improving production performance.

The LEfSe analysis further substantiated these shifts, identifying *Treponema*, *Succinatimonas*, and *Ruminococcus* as biomarkers of the *Verbena* group. *Treponema* is involved in fiber degradation (40), while *Succinatimonas* can ferment glucose and other carbohydrates to generate short-chain fatty acids, especially acetate and succinate, that can benefit enterocyte development (41). Moreover, *Ruminococcus* is involved in dietary fiber fermentation, producing butyrate, which plays a critical role in reducing inflammation and maintaining the health of the gut epithelium (42). These taxa suggest enhanced fermentation capacity, immunity, and microbial efficiency in ducks receiving *Verbena*.

Moreover, we further analyzed the changes in the levels of 10 brain metabolites related to neurotransmitter function. Supplementation with *Verbena* resulted in higher levels of Gln, while reducing the levels of GABA, Tyr, Glu, and Ach. In addition, supplementation with *Polygonum cuspidatum* increased the levels of GABA, Gln, and 5-HIAA while decreasing Glu and Ach. Resveratrol, a bioactive polyphenol found in *Polygonum cuspidatum*, has been shown to modulate GABAergic transmission by enhancing GABA receptor activity and exerting neuroprotective effects via antioxidant and anti-inflammatory mechanisms, including the activation of the Nrf2 pathway and the suppression of NF-κB signaling (43). These mechanisms can lead to the upregulation of endogenous antioxidant enzymes such as superoxide dismutase (SOD) and glutathione peroxidase (GPx), which help mitigate oxidative damage in neural tissues. Resveratrol has also been reported to reduce pro-inflammatory cytokines like TNF-*α* and IL-6, which are key mediators in neuroinflammation (22). Similarly, verbascoside, a phenylethanoid glycoside abundant in *Verbena*, has been reported to influence cholinergic signaling by increasing Ach levels and inhibiting acetylcholinesterase activity, which may enhance cognitive and neurobehavioral functions (44). However, in this study, *Verbena* supplementation led to decreased Ach, suggesting a context-dependent or dose-dependent modulation of the cholinergic system. This compound has also demonstrated nutrigenomic activity, potentially influencing gene expression related to oxidative stress and inflammation, including SOD, GPx, TNF-α, and IL-6, thereby contributing to neural homeostasis (45).

GABA and Glu are important inhibitory neurotransmitters in the central nervous system (46). GABA, in particular, is known to stimulate food intake in chicks via activation of the GABAergic system through GABA receptors (47). A reduction in GABA levels may therefore explain the observed changes in feeding behavior and reduced appetite in ducks during brooding. The concurrent increase in Gln may support intestinal health, as Gln serves as a primary energy substrate for enterocytes and helps maintain gut barrier integrity by reducing intestinal permeability (48). Ach, as the primary neurotransmitter, is associated with cognition, learning, and memory in the neuromuscular and sensory systems for most vertebrates. AchE plays a critical role in Ach degradation and regulating cholinergic nervous transmission (49). Importantly, these neurochemical changes may be mediated via the gut–brain axis, which enables bidirectional communication between the gut microbiota and the central nervous system. Changes in the cecal microbiota composition, such as the observed decrease in *Firmicutes* following *Verbena* supplementation, can influence microbial metabolite production, including short-chain fatty acids and tryptophan metabolites, which are known to impact brain function through vagal nerve signaling or systemic immune modulation. Inflammatory cytokines produced in response to gut dysbiosis may also cross the blood–brain barrier, influencing the synthesis and metabolism of key neurotransmitters. In addition, increased 5-HIAA, the main serotonin metabolite, in the *Polygonum cuspidatum* group suggests that this herb may enhance serotonin turnover. This aligns with the known effect of resveratrol (from *Polygonum cuspidatum*) on serotonergic pathways, including its antidepressant-like effects in mammals (50). This could have implications for mood regulation and stress resilience in ducks. Furthermore, the observed reductions in GABA and Tyr in the Verbena group may represent a form of metabolic rebalancing or stress modulation induced by the herb’s phytochemicals. Tyr is a precursor for catecholamines such as dopamine and norepinephrine, which are central to arousal and stress responses. A decrease in Tyr may therefore reflect reduced catecholaminergic activity, potentially indicative of a calmer or less stressed physiological state. Taken together, the alterations in brain metabolites observed in response to *Verbena* and *Polygonum cuspidatum* supplementation appear to be mediated by a combination of antioxidant and anti-inflammatory pathways, nutrigenomic effects, and gut–brain axis modulation. These changes likely contribute to improved neurophysiological balance, stress resilience, and possibly energy repartitioning in Sansui ducks. Further mechanistic studies, including gene expression analysis and microbial metabolite profiling, are warranted to fully elucidate these interactions and their implications for poultry performance and welfare.

Neurotransmitters are essential endogenous chemical messengers that govern fundamental nervous system functions, including communication via the brain-gut axis (51). As such, they can directly influence the activity and composition of the gut microbiota community (52). The correlation analysis demonstrates that, upon exposure to *Verbena* and *Polygonum cuspidatum*, 3 altered metabolites (GABA, Ach, and Glu) involved in neurotransmitter production and function were highly related to changes in the microbial groups, including *Veillonella*, *Anaerosporobacter*, *Anaerofustis*, *Fusobacterium*, *Flavonifractor*, *Intestinimonas*, *Peptoclostridium*, and *Lactobacillus*, as compared to the control group. *Veillonella* and *Anaerofustis* are known to produce short-chain fatty acids and lactate, which can influence neurochemical production in the gut epithelium and enteric neurons (53). Previous studies found that *Flavonifractor* produces butyrate through lysine fermentation, which can reduce intestinal inflammation and improve intestinal barrier function (40). In this study, Ach was positively correlated with *Flavonifractor. Lactobacillus* are well-studied for their capacity to produce GABA directly from glutamate, a phenomenon observed in both mice (54). The abundance of *Lactobacillus* is beneficial for strengthening the intestinal barrier by enhancing tight intestinal epithelial junctions (55), and relatively intact intestinal function should be beneficial for the absorption of nutrients. Previous studies have explored the interactions between brain metabolites, including neurotransmitters, and gut microbiota, such as *Proteobacteria*, *Firmicutes*, and *Bacteroidetes* (56). However, it remains unclear how *Verbena* and *Polygonum cuspidatum* target key bacteria and metabolites to regulate gut health, and these metabolic phenotypes’ changes need to be further explored in the mechanism (57). Future studies using microbiota-targeted interventions, such as fecal transplantation or germ-free models, are needed to confirm causal microbiota–brain metabolite relationships. On the other hand, without direct molecular validation, the mechanistic pathways linking microbiota to neurochemical changes cannot be fully established. Future studies employing transcriptomic or proteomic analyses of gut and brain tissues are necessary to elucidate signaling pathways and gene expression patterns involved in microbiota–brain communication (58, 59). Such multi-omics approaches would strengthen the understanding of how phytogenic interventions like *Verbena* and *Polygonum cuspidatum* influence host neurobiology through the gut–brain axis in the ducks.

## 5 Conclusion

To the best of our knowledge, this study is the first to establish a connection between dietary supplementation with *Verbena officinalis* and *Polygonum cuspidatum* and both the gut microbiota composition and brain metabolite profiles in ducks. Dietary supplementation with both herbal sources in Sansui ducks improves growth performance and immune response, modifies the composition of cecal microbiota, and alters the brain metabolite (GABA, Gln, Glu, Tyr, Ach, and 5-HIAA) profile. The strong correlations between gut bacterial taxa (*Veillonella*, *Anaerofustis*, *Fusobacterium*, *Flavonifractor*, and *Lactobacillus*) and brain metabolites (GABA, Ach, and Glu) suggest that these herbs may influence duck physiology through the gut-brain axis. These findings contribute novel insights into how dietary phytogenic feed additives can shape animal health beyond gut-level changes, potentially impacting behavior, stress response, and neural function in poultry. Moreover, this study is also distinguished by its integration of growth data, gut microbiome, and brain metabolite analysis, which uncovers a complex interplay between diet, gut ecology, and brain biochemistry. One limitation of this study is the absence of cecal metabolomic analysis, which restricts our ability to fully characterize the functional link between microbial composition and brain metabolites. While we observed distinct changes in microbiota and brain metabolic profiles, the lack of gut metabolite data limits mechanistic interpretation of microbiota–metabolite–brain interactions. Future studies integrating gut content metabolomics are needed to clarify the metabolic pathways involved in gut-brain communication. Additionally, incorporating behavioral assessments and stress biomarkers (e.g., corticosterone) would further strengthen the link between microbial shifts, brain function, and animal welfare outcomes. Nonetheless, our results offer important initial insights into how *Verbena* and *Polygonum cuspidatum* may influence host physiology via the gut-brain axis during early development.

## Data Availability

The raw 16S rRNA gene and metagenomic sequencing data are available at the NCBI Sequence Read Archive (SRA), under BioProject PRJNA1145929.

## Footnotes

^1^ https://qiime2.org/
